# Insect community in riparian zones of Sungai Sepetang, Sungai Rembau and Sungai Chukai of Peninsular Malaysia

**DOI:** 10.3897/BDJ.7.e35679

**Published:** 2019-09-18

**Authors:** Nur-Athirah Abdullah, Siti Nur Fatehah Radzi, Lailatul-Nadhirah Asri, Nor Shafikah Idris, Shahril Husin, Azman Sulaiman, Shamsul Khamis, Norela Sulaiman, Izfa Riza Hazmi

**Affiliations:** 1 Center of Ecosystem Management and Natural Resources, Faculty of Science and Technology, Universiti Kebangsaan Malaysia, 43600 Bandar Baru Bangi, Selangor, Malaysia Center of Ecosystem Management and Natural Resources, Faculty of Science and Technology, Universiti Kebangsaan Malaysia 43600 Bandar Baru Bangi, Selangor Malaysia; 2 Center for Insect Systematics, Faculty of Science and Technology, Universiti Kebangsaan Malaysia, 43600 Bandar Baru Bangi, Selangor, Malaysia Center for Insect Systematics, Faculty of Science and Technology, Universiti Kebangsaan Malaysia 43600 Bandar Baru Bangi, Selangor Malaysia; 3 Centre for Earth Sciences and Environment, Faculty of Science and Technology, Universiti Kebangsaan Malaysia, 43600 Bandar Baru Bangi, Selangor, Malaysia Centre for Earth Sciences and Environment, Faculty of Science and Technology, Universiti Kebangsaan Malaysia 43600 Bandar Baru Bangi, Selangor Malaysia; 4 TNB Research Sdn. Bhd, No. 1, Lorong Ayer Hitam, Kawasan Institut Penyelidikan, 43000, Kajang, Selangor, Malaysia TNB Research Sdn. Bhd No. 1, Lorong Ayer Hitam, Kawasan Institut Penyelidikan, 43000, Kajang, Selangor Malaysia

**Keywords:** Insect community, Insect-plant interaction, insect guild, plant community, Sungai Sepetang, Sungai Rembau, Sungai Chukai

## Abstract

Riparian areas hold vast number of flora and fauna with exceptional contributions to the ecosystem. A study was conducted in Sungai Sepetang, Sungai Rembau and Sungai Chukai to identify the insect community in a riparian zone of Peninsular Malaysia. Sampling was conducted in six consecutive months from December 2017 to May 2018 during both day and night using sweep nets. Twenty sampling stations (S1-S20) had been assembled along the riverbanks with an average distance of 200 m between each station. The 17,530 collected insects were from 11 orders and consisted of Diptera, Coleoptera, Hemiptera, Hymenoptera, Lepidoptera, Neuroptera, Orthoptera, Blattodea, Thysanoptera, Mantodea and Odonata. The three most abundant orders were Diptera (33.84%; 5933 individuals), Coleoptera (28.82%; 5053 individuals) and Hemiptera (25.62%: 4491 individuals). The collected insect community consisted of different guilds such as the scavenger, predator, herbivore, pollinator and parasitoid. Sungai Sepetang and Sungai Rembau were dominated by mangrove flora, *Sonneratia caseolaris* (Myrtales: Lythraceae), while Sungai Chukai was dominated by *Barringtonia racemosa*. There was a significant difference (p < 0.05) in the composition of insects between the three rivers though clustering analysis showed that the insect communities in Sungai Sepetang and Sungai Rembau were 100% similar compared to Sungai Chukai which consisted of a totally different community. There is a significant negative correlation between abundance of insects with salinity and wind speed at Sungai Chukai and Sungai Sepetang.

## Introduction

Insects are an important faunal group in terrestrial ecosystems where they play vital roles in stabilising the ecosystem. Based on an estimation of global species richness, there are about 5.5 million species of insects already recorded (Stork 2018). An insect may occupy multiple niches as a pollinator, predator, herbivore, parasite, as well as symbiont. It is important to note that insects live simultaneously creating a stable ecosystem dynamic. In response to any environmental change, each species will behave complementarily to one another, as in one species will increase while the other decreases (Schowalter 2016). This group of organism is also sensitive to changes in environment, thus making it possible to be used as an indicator for conservation planning (Kremen et al. 1993). As a sophisticated group of organisms, insects may adapt to diverse environments including aquatic, semi-aquatic and terrestrial habitat. They also inhabit the most extreme conditions on earth (hot springs, tundra, deserts). Nevertheless, certain individual species may only live in a limited dimension of space (Stewart et al. 2015).

The riparian area, an interface between land and water, becomes the habitat to a wide range of flora and fauna. The riparian vegetation apparently supports both aquatic and terrestrial insects especially in providing space to find food, mating partners, refuge, as well as a resting place (Herrera and Dudley 2003). Some riparian floras recorded as being found in Malaysia are *Sonneratia caseolaris*, *Hibiscus tiliaceus*, *Nypa fruticans*, *Acrotichum aureum*, *Areca cathechu*, *Oncosperma tigillarium* and *Ficus* sp. All of these types of vegetation were commonly found in riparian habitats and are associated with firefly in Peninsular Malaysia (Khoo et al. 2012; Wan Juliana et al. 2012) and Thailand (Prasertkul 2018). However, a different flora community was recorded in the riparian area in Sabah where there were *Rizophora apiculata*, *Clerodendrum inerme*, *Glochidion littorale*, *Bruguiera parviflora* and *Excoecaria indica* (Chey 2004; Dawood and Saikim 2016). Uniquely, one of the riparian vegetation, *Sonneratia caseolaris*, known as Berembang, was found to be the most preferable display tree by the synchronous firefly, *Pteroptyx tener* in Peninsular Malaysia (Jusoh et al. 2010; Wan Jusoh et al. 2010).

Firefly is a huge commodity for this country as it becomes a centre of attraction in the ecotourism industry. A spectacular flashing pattern is produced through light emitting reaction catalysed by Luciferase in the firefly abdomen (Nur Khairunnisa et al. 2016). The flashing light of firefly is captivating for the tourist visiting the riparian zone as its natural habitat. Multiple studies had been done in monitoring of the firefly population in riverbanks of Malaysia (Sulaiman et al. 2017; Hazmi and Sagaff 2017; Foo and Dawood 2017). However, there is a limited amount of research being undertaken in recording other insect communities coexisting in the same riparian area. It is advantageous to have knowledge of other insect populations living in the same riparian zone as an effort for firefly conservation, as well as the entire riparian ecosystem. We want to emphasise the need to study insect communities besides firefly, thus the data on firefly were not included in this study. Hence, this study is a preliminary assessment done to 1) identify the insect community in the riparian zone, 2) identify riparian vegetation in which the insect community resides and 3) determine changes in the insect community across several environmental parameters (salinity, wind speed, temperature and humidity).

## Materials and Method

**Figure 1.**
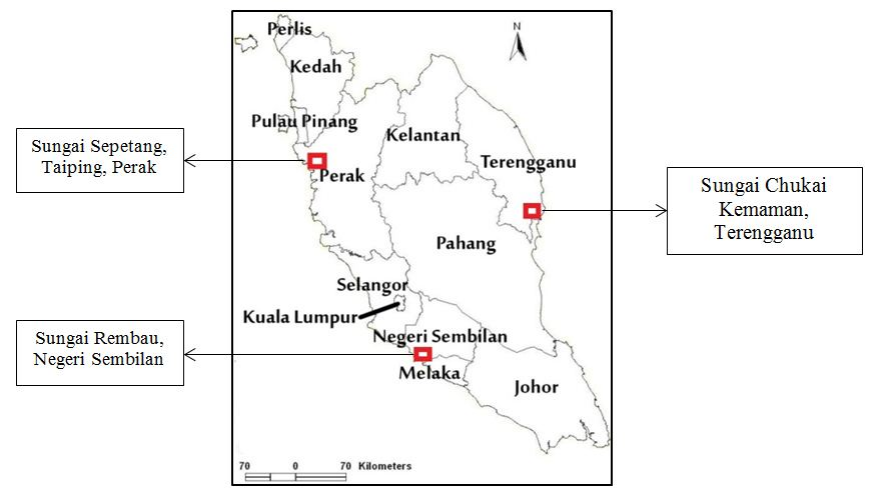
Location of the study sites in Peninsular Malaysia.

Samplings were conducted in 20 sampling stations along the riverbanks of Sungai Sepetang, Taping, Perak, (Lat 4.8866–4.9092ºN, Lon 100.6311–100.6648ºE), Sungai Rembau, Negeri Sembilan (Lat 2.4191–2.4484ºN, Lon 102.0654ºE) and Sungai Chukai, Kemaman, Terengganu (Lat 4.3076–4.3002ºN, Lon 103.3725–103.395ºE); see Fig. 1. At each locality, twenty riparian trees along the river with an average distance of 200 m were selected as sampling stations. The sampling stations were assembled at both sides of the riverbanks as shown in Fig. 2. They were labelled as S1- S20 where S1 was located closest to the downstream while S20 was furthest. Samples of insects were collected monthly for six consecutive months (December 2017 to May 2018) during day and night using sweep nets. The net was swept for one minute at each sampling station in the study area. Insect samples were placed in bottles containing ethyl acetate and were then brought to the laboratory for identification up to the lowest taxonomic level possible. Each station was observed from the boat to record the riparian tree and others species composition located within a 5 m radius from the sampling station. Abiotic factors such as temperature (°C), relative humidity (%), salinity (% of NaCl) and wind speed (m/s) were also recorded at each sampling station. Environmental temperature and relative humidity were measured using a thermo hygrometer HI8564 by Hanna Instruments. Salinity was measured using a NaCl meter HI 9835 by Hanna Instruments, while wind speed was measured using an anemometer PCE-007 by PCE instruments.

**Identification of specimens**

The insect specimens were identified to the family level using the key published in Triplehorn and Johnson 2005. All specimens were also compared to the collection of insects in the insect repository of Center of Insect Systematics, The National University of Malaysia. If it were not possible to determine the species, insect specimens were then classified into morphospecies. The specimens collected were then deposited in the Center for Insect Systematics, Universiti Kebangsaan Malaysia.

## Data Analysis

Data assessed for normality by the Shapiro–Wilk test (p < 0.05) were found to be not normally distributed. Therefore, a non-parametric Kruskall–Wallis test was run to determine whether differences occurred to insect communities in different locations. Spearman’s correlation analysis was used to test the correlation between abundance and diversity of insect communities and abiotic factors (salinity, wind speed, temperature and relative humidity). ANOVA is used to test for significant differences of abiotic factors between each study location. The Shapiro-Wilk test was performed using R 3.5.3 while the Kruskall-Wallis test, correlation analysis and ANOVA were performed using Minitab 17. Species diversity was determined by using the Shannon Diversity Index (H’). Cluster analysis, using the Sørensen-Bray Curtis distance measure, was carried out to find the overlapping of the insect community at three different locations. Insect families with a presence of less than five individuals were identified as outliers and excluded from cluster analysis. The PCORD programme (MjM Software, Oregon 2001) was used for both diversity index and cluster analysis.

## Results and Discussion

**Insect and vegetation spatial variability across different locations**

**Figure 3.**
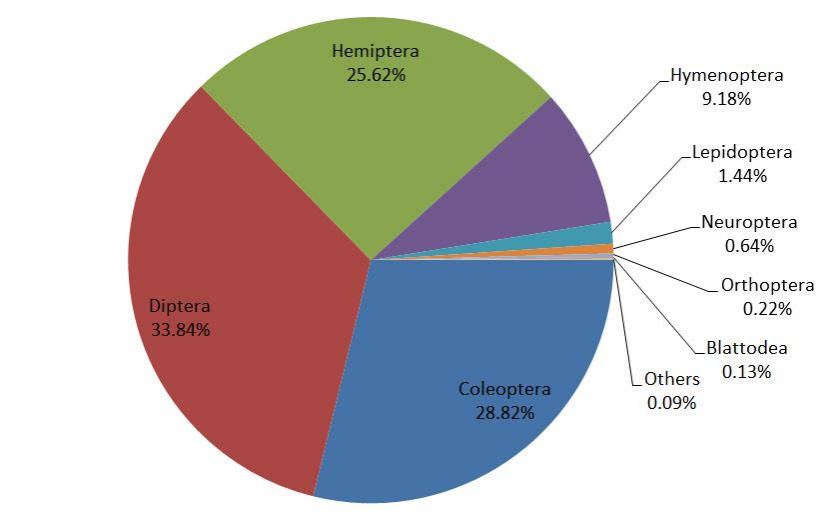
Composition of insect order in riparian zones of Peninsular Malaysia.

A total of 17,530 insect specimens were collected in six consecutive months from three riparian zones in Peninsular Malaysia. The collection consisted of 11 insect orders consisting of Diptera, Coleoptera, Hemiptera, Hymenoptera, Lepidoptera, Neuroptera, Orthoptera, Blattodea, Thysanoptera, Mantodea and Odonata. Three most abundant orders are Diptera (33.84%; 5933 individuals), Coleoptera (28.82%; 5053 individuals) and Hemiptera (25.62%: 4491 individuals) (Fig. 3). There is a significant difference (*H* = 11.83; df = 2; p < 0.05) between the insect composition in the three locations. Nevertheless, clustering analysis provided an insight as to how the insect community differed between locations.

**Figure 4.**
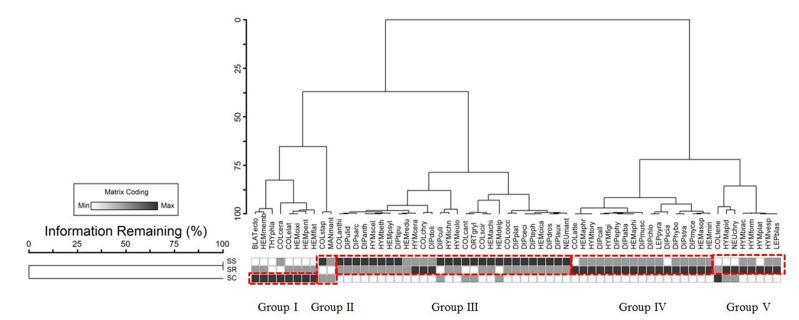
Dendogram from two-way cluster analysis of insects in Sungai Sepetang-SS, Sungai Sungai Rembau-SR and Sungai Chukai-SC

The insect community in Sungai Sepetang and Sungai Rembau were 100% similar, while the community in Sungai Chukai was entirely different from the other two sites (Fig. 4). The insects collected in this study consisted of diurnal, nocturnal and crepuscular insects. Through clustering analysis, the insect community collected in this study can be categorised into four groups of different guilds at 75% overlapping value. Group I consisted of diurnal herbivorous insects. Generalist insects, which occupy a wide range of sources, were clustered into Group II. This group consisted of herbivores, predators and scavengers that are active both diurnally and nocturnally. Group III consisted of diurnal predators, herbivores as well as scavengers, while Group IV consisted of diurnal scavenging flies. The community of insects in Sungai Chukai were characterised by the absence of these scavenging flies along with several families of gall-inducing insects and leaf rollers, such as Aprophoridae, Aphididae and Attelabidae with parasitoid groups such as Figitidae and Torymidae. A checklist of insects collected in the riparian zones of Peninsular Malaysia is shown in (Table 1).

**Figure 5.**
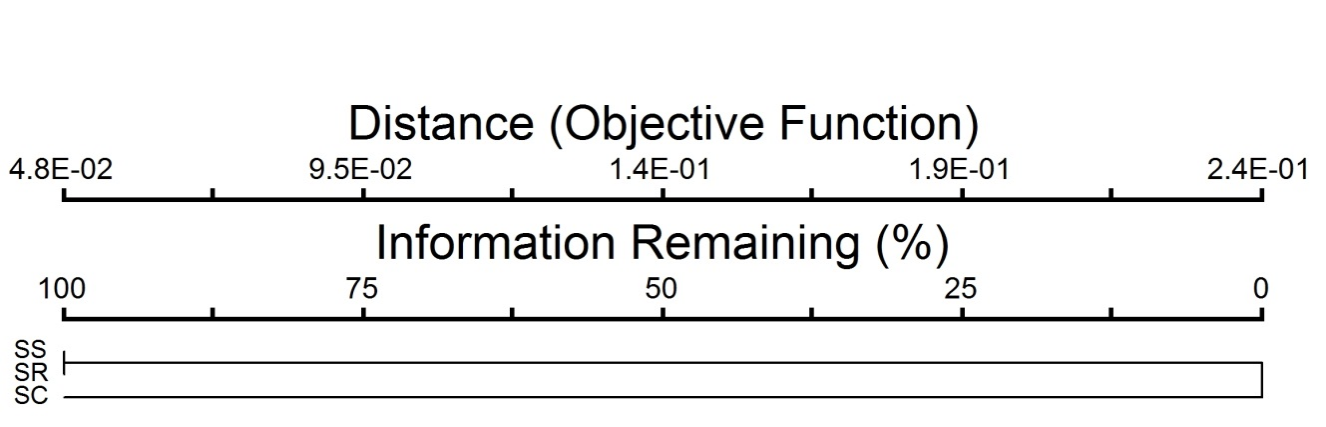
Dendogram from one way cluster analysis for vegetation found at Sungai Sepetang-SS, Sungai Rembau- SR and Sungai Chukai-SC.

The variation of insect communities in Sungai Sepetang, Sungai Rembau and Sungai Chukai is strongly due to the difference in vegetation composition at each location. A total of 16 flora species were recorded during the observation at each sampling station in all locations. The list of flora species found in each site is shown in Table 2. One-way cluster analysis showed that the vegetation compositon in Sungai Chukai is distinct from the vegetation composition in Sungai Sepetang and Sungai Rembau which have a 100% similarity (Fig. 5). In this study, the riverbank of Sungai Chukai is highly dominated by mangrove associates *Barringtonia racemosa*, while both Sungai Sepetang and Sungai Rembau are dominated by the true mangrove, *Sonneratia caseolaris.* Sungai Chukai also recorded the presence of other mangrove associates, such as *Oncosperma tigillarium*, *Vitex pinnata*, *Brugeira sexangula* and *Melastoma malabathricum* which were absent in the other two locations. Since the vegetation composition is different between Sungai Chukai and the other two locations, it then brought different communities to reside within it. The same result was also reported by Veenakumari and Prashanth (2009), where the true mangrove plant, such as *Sonneratia* and *Rizophora*, harbours a different entomofaunal complex when compared to the mangrove associates, such as *Barringtonia* and *Hibiscus*.

The diversity of vegetation in Sungai Chukai is 1.998 when compared to Sungai Rembau (H’ = 1.816) and Sungai Sepetang (H’ = 1.591). In coherence with this situation, the insect diversity is recorded to be highest in Sungai Chukai (H’ = 3.831). The diversity of insects in Sungai Rembau is 3.599 and lowest in Sungai Sepetang (H’ = 3.398). The result supports a positive correlation between plant diversity and insect diversity (Hunter and Price 1992; Morante-Filho et al. 2016). The vegetation structure is a large deciding factor for the insect assemblage presence at each site (Schaffers et al. 2008). Every type of vegetation will have a unique quantity and quality of resources. Variation in quantity and quality of resources between the plant species may be reflected by looking at the plant structure (Randlkofer et al. 2010) and leaf quality (Barber and Marquis 2011), as well as its chemical attributes (Joern and Laws 2013). The chemical content of a plant may act as insect attractants (Yu et al. 2008) or repellants (Maia and Moore 2011). Therefore, we suggest for the study of phytochemical and tree architecture of plant species in the riparian zone of Peninsular Malaysia to be done for a more comprehensive understanding with regards to this matter.

In comparison to the high species richness of insects in Sungai Chukai, the abundance of insects was shown to be lowest out of all three locations. Apparently, *B. racemosa* that dominates the riverbank of Sungai Chukai have insecticidal properties, known as Saponin, which may repel the presence of insects (Izzati Osman et al. 2017). Besides that, the branching structure of *B. racemosa* is simpler when compared to the complex ramification of *S. caseolaris* that dominated both Sungai Sepetang and Sungai Rembau. According to Obermaier et al. 2008), each plant has a different structural architecture which may regulate insects' establishment in an area which is shown in this study. A complex structure of vegetation such as *S. caseolaris* will provide more resources in terms of leaf and litter for the insects to survive on, either as refugees or food source. This is supported by a study done by Li et al. (2012) which highlighted a significant difference in richness and abundance of ground-dwelling arthropods under different species of shrubs. A complex and larger-sized plant may serve as a greater source of feeding area and shelters (Stapp 1997). It is therefore important to take note of the key plant species in the effort of conservation management. This study managed to identify *S. caseolaris* as the key plant species in study sites, as it dominated the riverbanks and supports a wide variety of insects including the invaluable firefly which can be considered as an umbrella species to the area.

**Utilisation of resources by insect community**

The abundance of insects is very closely related to the quantity of resources available which includes food resources, habitat, mating partners and others. Following cluster analysis, phytophagous insects prevail in the riparian zone. These insects comprised of various orders, such as the Coleoptera, Hemiptera and Lepidoptera, as well as Orthoptera. These orders are known to cause damage to plants in mangrove areas. The true mangrove plants, such as *Sonneratia*, are prone to attack by sap feeders, such as Psyllidae, Membracidae (*Tricentrus* sp.), Flatidae (*Salurnis* sp.) and Cixiidae (Murphy 1990). It was also reported by Murphy (1990) that the Chrysomelid beetle was not a significant foliage grazer of *Sonneratia*. However, our study found substantial numbers of *Monolepta* sp. suggesting possible damage to the tree, caused by this insect family. This is especially true for *S. caseolaris* found in Sungai Chukai that depicts the obvious physical damage to the leaf, affecting its vigour. A further study needs to be undertaken to confirm this circumstance as to whether indeed the Chrysomelid beetle feeds on the leaf of *S. caseolaris* in Sungai Chukai. *Sonneratia* can also be affected by the Pyralidae moth which was also collected in abundance during this study. According to Murphy (1990) and Veenakumari et al. (1997), the Pyralidae moth might destroy both old and young leaves as well as bore into the fruit and flower of *Sonneratia*. This should raise concern about the effort of firefly conservation management. The health of *S. caseolaris* is crucial in maintaining firefly populations in the riparian area since the fireflies are highly associated with this mangrove species (Ayub et al. 2017). Hence, in an effort to maintain the firefly population, it is important to identify all possible factors that may cause damage to its display tree.

Nevertheless, Diptera is recorded to have high abundance at all three sites, especially in Sungai Rembau. Diptera is indeed one of the dominant orders amongst others, as it can be found in abundance within various ecosystems (Daly et al. 1998). In addition to the natural condition of the sampling area where riverbanks are subjected to water tides making it moist and humid for development of Dipteran larva, the high abundance of Diptera may also be caused by human activity. During the period of the study, there were multiple land uses next to the riverbank. In fact, human activities such as horticulture and livestock farming were common; oil palm plantation, cattle and a chicken farm were found close to the sampling station in Sungai Rembau. Apparently, the minimum buffer zone of 20 m on both sides of the riverbank were exploited for such activities. The minimum buffer zone of 20 m was proposed in 2016 to overcome water pollution problems (Lokman 2016). However, the regulations were infringed as oil palm plantation areas and a livestock farm were erected up to the water edge. Livestock farming such as a cattle farm and a chicken farm in Sungai Rembau may contribute to the high number of scavenging fly individuals which have been attracted to the animal waste. Besides that, the high abundance of Diptera were fixed upon the natural habitats for Chironomidae, Ephydridae and Hybotidae which were all commonly found in shores and marsh areas (Grootaert and Shamshev 2012, Triplehorn and Johnson 2005). There is a strong presence of *Acropsilus* sp., *Drosophila* sp. as well as *Hardyadrama* sp. on the other hand, due to the rotten *S. caseolaris* fruits that become their source of food.

The predator and parasitoid insect groups were also recorded in the riparian zone of our study. This insect group assists in stabilising the insect community in the riparian zone by keeping the population of herbivorous insects at an optimum level. Parasitoid wasps, such as Bethylidae, *Goniozus* sp., Braconidae; *Cotesia* sp., *Phanerotoma* sp., Ceraphronidae; *Ceraphron* sp., Eulophidae; *Euplectrus* sp. and Ichneumonidae; *Arhytis* sp. are known to have a beneficial use in agriculture, such as oil palm. *Cotesia* sp., for instance, is an important natural enemy to bagworm (Psychidae; *Metisa plana)*, an important pest to the oil palm plantations of Peninsular Malaysia (Halim et al. 2018). As the location of study sites in Sungai Rembau and Sungai Sepetang are located next to oil palm plantations, these beneficial insects may move across the boundaries and colonise the adjacent plantations and then exert their beneficial effects upon the pests in the agricultural plantations. The predator group, such as Reduviidae, also acts the same way as the parasitoid wasps in maintaining a balanced ecosystem. It is also important to note that Reduviidae might take firefly species as a prey. A species of the assassin bug, *Zelus luridus* has been observed to feed on firefly species, *Photinus carolinus* (Lewis et al. 2012). This ecological relationship should be highlighted in the process of firefly conservation.

It is interesting to document a family of soldier beetle, Cantharidae in this study as *Cantharis* sp. and *Pacificanthia* sp. seem to resemble the morphological character and colouration of the firefly, *Pteroptyx tener*. There is a potential mimicry of the soldier beetle to the firefly species, based on this encountered record. The soldier beetle of different species was also found to mimic other species of firefly, *Pteroptyx effulgens* in Papua New Guinea (Ohba and Meyer-Rochow 2012). Cantharidae is also involved in the mimicry complexes with fireflies as recorded by Lloyd 1973. A possible explanation to this occurrence is of Batesian mimicry with the basic principle that the mimics must be less common compared to the model (Ohba and Meyer-Rochow 2012). As the firefly is dominant in the riparian zone of our study area, it easily outnumbered the other co-existing insects including the soldier beetle. However, as the Cantharids are predatory, the more likely situation would be of Mertensian mimicry. Mertensian mimicry occurs when the deadly species mimics the harmless species as a lesson-teaching model (Emsley 1966). It is hypothesised through this finding that Cantharid beetle mimics the firefly in order for it to look innocuous to other prey insects residing on the same vegetation in the daytime. There is also an additional advantage in the lesson-teaching model as a higher predator to the cantharid beetle could learn to avoid them from the unpalatable firefly. The likelihood of mimicry between the soldier beetle and the congregating firefly is a new finding that needs to be explored through further ethological and ecological study.

**Environmental influences over insect community**

The average value of wind speed (m/s), salinity (% of NaCl), temperature (⁰C), relative humidity (RH%) are shown in Table 3. The Spearman’s correlation values are summaried in Table 4.

The salinity at each sampling point in all locations decreased with an increase in distance from the outfall. There is a significant negative correlation between abundance of insects in Sungai Chukai with salinity. The diversity of insects in Sungai Sepetang also negatively correlated with the salinity of the river. The total abundance of insects in Sungai Sepetang, Sungai Rembau and Sungai Chukai comparatively showed an increasing trend as salinity decreased from S1 to S20 (Fig. 6). The salinity of the water is especially critical for the growth of aquatic and semi-aquatic insects including the firefly population. Results from this study showed a relative pattern at which the abundance of insects is increasing with the decreasing value of salinity. As most of the larval growth occurred in water, salinity of the river needs to be within a tolerated range. An increase in salinity of the river is an issue faced by several insects, such as the mayflies and non-biting midges, *Chironomus* sp. (Hassell et al. 2006). The salinity increase caused the growth rate of the insect to be slower and reduced the number of emerging adult (Castillo et al. 2018; Kefford et al. 2006). Salinity may also affect the soil where fireflies laid their eggs and hatch as larvae though the direct effect of salinity on the firefly has never been studied. Nonetheless, Khoo et al. 2012 reported that the sudden increase in salinity in Sungai Selangor does not affect the firefly population residing on *S. caseolaris* at the riverbanks. In spite of that, it is still essential to monitor the river water salinity in our study area in order to oversee the health of these rivers as a water supply to the whole riparian ecosystem.

There is also a significant negative correlation between abundance and diversity of insects in Sungai Sepetang with wind speed. Although temperature and humidity show no significant correlation to the insect community, a hotter and less humid condition can cause death to the insects. Riparian vegetation acts as a key role in determining riparian microclimatic conditions (Briers and Gee 2004). Temperature, humidity, light intensity and wind speed are very much affected by vegetation type and lushness. The canopy of the tree provide shades from light which will also then regulate river water temperature (Garner et al. 2017). This vegetation also acts as a windbreak and reduces the force of air towards the soil (Okin 2008), as well as to the small-bodied insect species living in the area (Pasek 1988). While the movement of air helps in flying insect movement, a high speed of wind may affect small-sized insects in directing them to less optimal directions as they are easily carried away by the fast moving air (Gatehouse 1997). Based on the size of insect individuals ranging from 0.1 mm to 5.0 mm collected from this study, it is very much expected for them to be affected by wind speed. For that reason, it is important for us to highlight the importance of conserving the riparian zones as a whole, though it proved to be difficult when facing many land use changes. This can be significantly seen in Sungai Rembau and Sungai Sepetang where the riparian areas are rapidly converted into agricultural, industrial and residential areas. The study of land use changes in our study sites is currently ongoing to further verify this issue. For the time being, this study sees the need for tighter regulations in maintaining riparian zones that will help to conserve the insect communities living within it.

## Conclusion

We have successfully made a list of insect taxa in riparian zones of Peninsular Malaysia. The list may serve as a solid baseline to compare with future data. In the light of firefly conservation as a unique inhabitant of the study areas, it is also important to understand how this insect community interacts with the firefly population. This question needs to be answered with future studies for the whole ecosystem knowledge to be expanded. Nonetheless, at the present moment, we insist upon and anticipate a more dynamic effort in the conservation of the riparian area. A conservation effort, especially for the vegetation community, is much needed as it holds the key to the maintenance of the riparian ecosystem as a whole, including the insect community that dwells within it.

## Figures and Tables

**Figure 2a.**
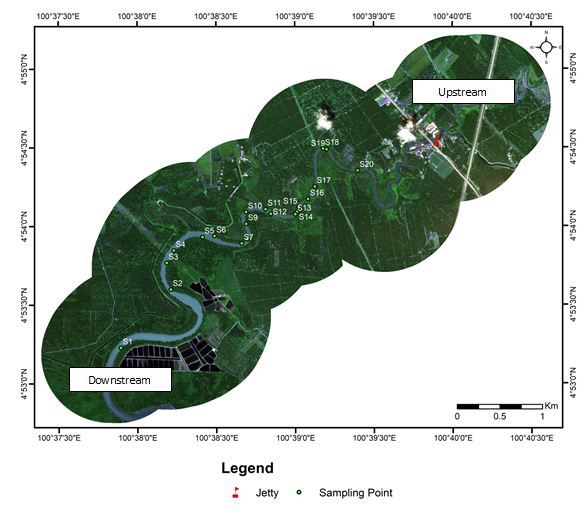
Sungai Sepetang, Taiping, Perak

**Figure 2b.**
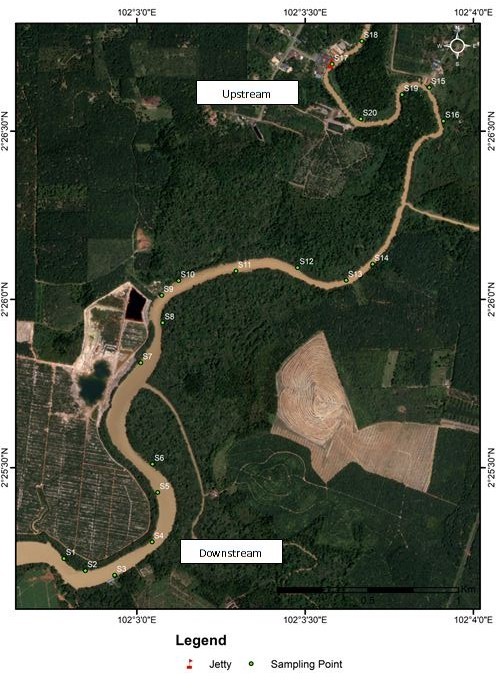
Sungai Rembau, Linggi, Negeri Sembilan

**Figure 2c.**
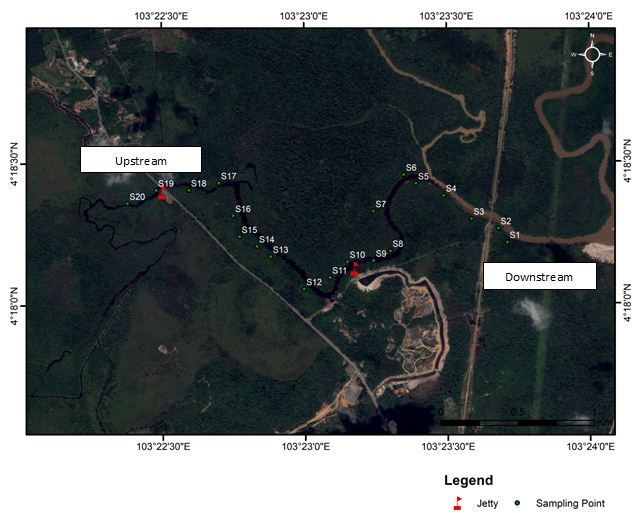
Sungai Chukai, Kemaman, Terenggan

**Figure 6a.**
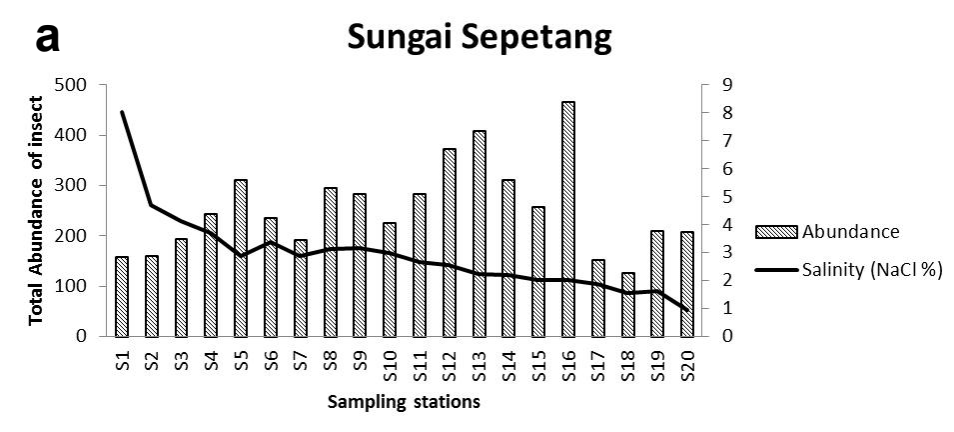
Sungai Sepetang, Taiping, Perak

**Figure 6b.**
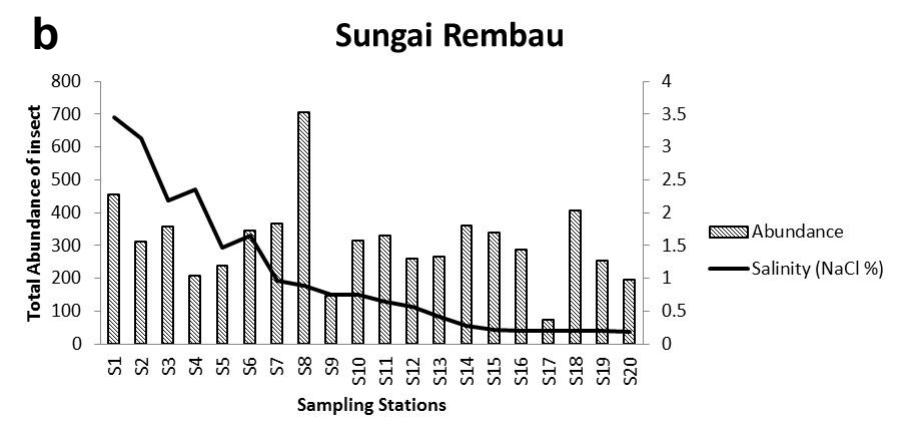
Sungai Rembau, Linggi, Negeri Sembilan

**Figure 6c.**
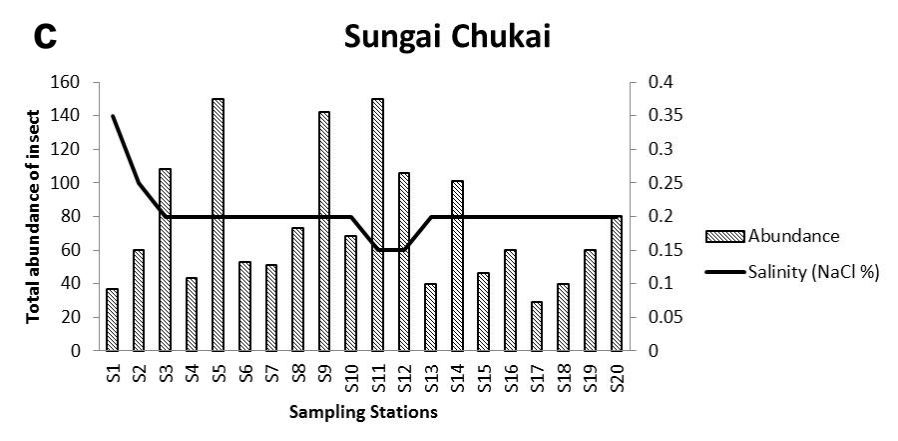
Sungai Chukai, Kemaman, Terenggan

**Table 1.** List of insect fauna in riparian zones of Sungai Sepetang, Sungai Rembau and Sungai Chukai, Peninsular Malaysia

**Order**	**Family**	**Sub-family**	**Species**	**Sungai Sepetang**	**Sungai Rembau**	**Sungai Chukai**
** Blattodea **						
	** Ectobiidae **		Ectobiidae gen. sp.			●
		Anaplectinae	*Anaplecta* sp.	●		
		Blattellinae	*Blatella germanica*		●	●
			*Symploce* sp.	●	●	
** Coleoptera **						
	** Anthicidae **	Anthicinae	*Anthicus* sp.1	●	●	
			*Anthicus* sp.2	●	●	●
		Macratriinae	*Macratria* sp.		●	
	** Anthribidae **	Anthribinae	*Acorynus* sp.	●		
	** Attelabidae **	Rhynchitinae	*Auletobius* sp.		●	
	** Buprestidae **	Agrilinae	*Agrillus* sp.	●	●	
	** Cantharidae **	Cantharinae	*Cantharis* sp.	●	●	●
			*Pacificanthia* sp.		●	
		Silinae	*Silis* sp.1	●		
			*Silis* sp. 2	●		
	** Carabidae **	Lebiinae	*Ophionea indica*	●		
	** Cerambycidae **	Cerambycinae	*Ceresium furtivum*	●		●
	** Chrysomelidae **	Eumolpinae	*Basilepta multicostata*		●	●
			*Rhyparida wallacei*	●	●	
			*Tricliona* sp. 1	●	●	●
			*Tricliona* sp. 2	●		
		Galerucinae	*Arthrotus capitata*	●	●	
			*Dercetisoma* sp.	●	●	●
			*Mimastra sp.*	●	●	
			*Monolepta bifasciata*	●	●	●
			*Monolepta rufipennis*	●	●	●
			*Monolepta terminata*		●	●
			*Palpaenidae pallipes*	●		●
	** Coccinellidae **	Chilocorinae	*Brumus* sp.			●
			*Exochamus aethiops*	●		
		Epilachninae	*Henosepilachna* sp.		●	
		Coccinellinae	*Heteroneda reticulata*	●	●	●
	** Curculionidae **	Cryptorhynchinae	*Rhadinomerus* sp.	●		
		Scolytinae	*Coccotrypes* sp.	●	●	
	** Elateridae **	Esthesopinae	*Priopus* sp.			●
		Agrypninae	*Pyrophorus* sp.		●	
	** Lampyridae **	Luciolinae	*Pteroptyx tener*	●	●	●
			*Pteroptyx malaccae*	●	●	
			*Pteroptyx asymmetria*	●		
			*Pteroptyx valida*			●
	**Scarabaedae**	Rutelinae	*Adoretus sinicus*	●		●
	** Scirtidae **	Scirtinae	*Contacyphon* sp.	●	●	●
			*Nyholmia collaris*	●	●	●
			*Ora* sp.	●	●	●
			*Scirtes flavoguttatus*	●	●	●
			*Scirtes* sp.1	●	●	●
			*Scirtes* sp.2		●	
			*Scirtes* sp.3			●
			*Scirtes* sp.4	●	●	
			*Scirtes* sp.5	●	●	●
	** Staphylinidae **	Paederinae	*Paederus* sp.	●		
			*Pinophilus* sp.	●		
		Oxytelinae	*Carpelimus* sp.	●		●
	** Tenebrionidae **	Lagriinae	Lagriinae gen sp.			●
			*Cerogria* sp.	●	●	●
** Diptera **						
	** Calliphoridae **		Calliphoridae gen. sp.	●	●	
	** Cecidomyiidae **		Cecidomyiidae gen. sp.		●	
	** Culicidae **	Anophelinae	*Anopheles* sp.	●	●	●
		Culicinae	*Armigeres* sp.	●	●	●
			*Culex* sp.	●	●	●
	** Chloropidae **		Chloropidae gen. sp. 1		●	●
			Chloropidae gen. sp. 2			●
		Oscinellinae	*Gaurax* sp. 1	●	●	●
			*Gaurax* sp. 2		●	●
	** Dolichopodidae **	Medeterinae	*Acropsilus* sp.	●	●	●
			Medeterinae gen. sp. 1	●	●	●
			Medeterinae gen. sp. 2	●	●	
	** Drosophilidae **	Drosophilinae	*Drosophila melanogaster*	●	●	●
	** Hybotidae **	Tachydromiinae	*Drapetis parillis*	●	●	●
			*Elaphropeza* sp.	●	●	
	** Ephydridae **	Discomyzinae	*Ceropsilopa* sp.	●	●	●
		Gymnomyzinae	*Discocerina obscurella*	●	●	●
			*Allotrichoma alium*	●	●	●
	**Faniidae**	Faniidae gen. sp.	Faniidae gen. sp.		●	
	** Lauxaniidae **		Lauxaniidae gen. sp.	●	●	
	** Muscidae **	Atherigoninae	*Atherigona* sp.	●	●	
		Coenosiinae	*Limnophora* sp. 1	●	●	●
	** Mycetophilidae **	Mycetophilinae	*Epicypta* sp.	●	●	
	** Platystomatidae **		Platystomatidae gen. sp.	●	●	
		Platystomatinae	*Scholastes* sp. 1	●		
			*Scholastes* sp. 2	●	●	
	** Sarcophagidae **		Sarcophagidae gen. sp.	●	●	
		Sarcophaginae	*Sarcophaga* sp.	●	●	
	** Sciaridae **	Cratyninae	*Bradysia* sp.	●	●	●
	** Sepsidae **		Sepsidae gen. sp.	●		
	** Stratiomyidae **		Stratiomyidae gen. sp.	●	●	
		Pachygastrinae	Pachygastrinae gen. sp.	●	●	
	** Tabanidae **	Tabaninae	*Tabanus* sp.	●	●	
	** Tephritidae **	Tephritinae	Tephritinae gen. sp.		●	
		Trypetinae	*Hardyadrama* sp.		●	
	** Tipulidae **		Tipulidae gen. sp. 1	●	●	●
			Tipulidae gen. sp. 2	●	●	●
			Tipulidae gen. sp. 3	●	●	
	** Ulidiidae **		Ulidiidae gen. sp.	●	●	
** Hemiptera **						
	** Achilidae **	Achilinae	Plectoderini gen. sp. 1	●	●	●
			Plectoderini gen. sp. 2	●	●	●
			Plectoderini gen. sp. 3	●		●
	** Aphalaridae **		Aphalaridae gen. sp.	●	●	●
		Spondyliaspidinae	Spondyliaspidinae gen. sp. 1	●	●	●
			Spondyliaspidinae gen. sp. 2	●	●	●
	** Aphididae **		Aphididae gen. sp	●	●	
	** Aphrophoridae **		Aphrophoridae gen. sp.	●	●	
	** Cicadellidae **	Typhlocybinae	Typhlocybinae gen. sp. 1	●	●	●
			Typhlocybinae gen. sp. 2	●		●
	** Cixiidae **		Cixiidae gen. sp. 1		●	
			Cixiidae gen. sp. 2		●	●
			Cixiidae gen. sp. 3		●	●
			Cixiidae gen. sp. 4			●
			Cixiidae gen. sp. 5			●
	** Delphacidae **		Delphacidae gen. sp		●	●
	** Dictyopharidae **	Dictyopharinae	Dictyopharinae gen. sp		●	
	** Flatidae **	Flatinae	*Siphanta* sp.		●	●
			*Salurnis* sp.			●
	** Hydrometridae **	Hydrometrinae	*Hydrometra* sp.			●
	** Membracidae **	Centrotinae	*Tricentrus* sp. 1	●	●	●
			*Tricentrus* sp. 2		●	●
			*Tricentrus* sp. 3	●		
			*Gargara* sp.			●
	** Miridae **		Miridae gen. sp.		●	●
		Bryocorinae	*Randallopsalus* sp. 1		●	●
			*Randallopsalus* sp. 2	●	●	
			*Helopeltis* sp.		●	
			*Felisacus* sp.		●	●
			*Michailocoris* sp.		●	
		Phylinae	*Pilophorus* sp.	●	●	●
	** Pentatomidae **		Pentatomidae gen. sp. 1		●	
		Asopinae	*Podisus* sp.	●	●	
		Pentatominae	*Nezara viridula*	●	●	●
	** Psyllidae **	Psyllinae	*Cacopsylla* sp.	●	●	●
		Spondyliaspidinae	*Boreioglycaspis* sp.		●	●
	** Pyrrhocoridae **	Pyrrhocorinae	*Dysdercus decussatus*			●
	** Reduviidae **	Emesinae	Emesinae gen. sp.			●
		Harpactorinae	*Isyndus heros*			●
			*Endochus* sp.		●	●
		Salyavatinae	*Lisarda inornata*			●
	** Scutelleridae **	Scutellerinae	*Calliphara nobilis*	●	●	
	** Tingidae **	Tinginae	*Stephanitis* sp.		●	
** Hymenoptera **						
	** Andrenidae **		Andrenidae gen. sp.	●	●	
	** Apidae **	Apinae	*Apis dorsata*		●	
		Xylocopinae	*Xylocopa latipes*			●
	** Bethylidae **	Bethylinae	*Goniozus* sp. 1	●	●	●
			*Goniozus* sp. 2	●	●	
	** Braconidae **		Braconidae gen. sp.		●	
		Cheloninae	*Phanerotoma* sp. 1	●	●	
			*Phanerotoma* sp. 2	●	●	●
			*Phanerotoma* sp. 3	●	●	●
			*Phanerotoma* sp. 4	●	●	●
		Doryctinae	Doryctinae gen. sp.		●	●
		Mesostoinae	Mesostoinae gen. sp.	●	●	●
		Microgastrinae	*Cotesia* sp.		●	●
	** Ceraphronidae **		Ceraphronidae gen. sp.	●	●	●
			*Ceraphron* sp.1	●	●	●
			*Ceraphron* sp.2	●		
	** Chalcididae **	Chalcidinae	*Brachymeria minuta*		●	
	** Crabronidae **	Pemphredoninae	Psenini gen. sp.	●		
	** Eulophidae **		Eulophidae gen sp. 1		●	
			Eulophidae gen. sp. 2	●	●	●
		Eulophinae	*Euplectrus* sp.	●	●	●
	** Evaniidae **		*Evania appendigaster*		●	
	** Figitidae **		Figitidae gen. sp.	●	●	
	** Formicidae **		Formicidae gen. sp. 1	●		
			Formicidae gen. sp. 2	●		
		Dolichoderinae	*Dolichoderus* sp.	●	●	●
		Formicinae	*Anoplolepis* sp.			●
			*Camponotus festinus*			●
			*Camponotus* sp.	●	●	
			*Euprenolepis* sp. 1	●	●	●
			*Euprenolepis* sp. 2	●	●	●
			*Formica* sp.			●
			*Oecophylla smaragdina*			●
			*Paraparatrechina* sp.	●		
			*Paratrechina* sp.	●		
			*Plagiolepis* sp.	●	●	
			*Polyrhachis furcata*	●		●
		Myrmicinae	Myrmicinae gen. sp.	●		
			*Myrmecina* sp.	●	●	
			*Crematogaster* sp.		●	●
			*Crematogaster claudiae*	●	●	●
			*Crematogaster rogenhoferi*			
			*Solenopsis* sp.	●	●	●
		Pseudomyrmecinae	*Tetraponera allaborans*	●		●
			*Tetraponera extenuata*	●	●	
	** Halictidae **	Nomiinae	*Nomia* sp.		●	
	** Ichneumonidae **	Banchinae	Banchinae gen. sp. 1	●	●	●
			Banchinae gen. sp. 2	●		
		Gelinae	*Arhytis* sp.	●	●	●
			*Messatoporus* sp	●	●	●
		Labeninae	Labeninae gen. sp.	●		
		Pimpilinae	*Xanthopimpla stemmator*	●		
		Tryphoninae	Tryphoninae sp.	●		
	** Mymaridae **		*Polynema* sp.			●
	** Perilampidae **	Perilampinae	Perilampinae gen. sp.	●	●	
	** Platygastridae **	Scelioninae	*Macroteleia* sp.			●
	** Scelionidae **	Telenominae	*Telenomus* sp.	●	●	●
	** Torymidae **	Megastiminae	*Megastigmus* sp.	●	●	
	** Vespidae **		*Donilus orientalis*	●		
		Polistinae	*Ropalidia malayana*	●	●	
			*Ropalidia sumatrae*	●	●	●
** Lepidoptera **						
	** Blastobasidae **		Blastobasidae gen. sp.	●	●	●
	** Pyralidae **		Pyralidae gen. sp.	●	●	●
** Mantodea **						
	** Mantidae **	Mantinae	Mantinae gen. sp. 1	●		●
			Mantinae gen. sp. 2			●
			Mantinae gen. sp. 3	●		
** Neuroptera **						
	** Chrysopidae **	Chrysopinae	*Chrysoperla* sp. 1		●	●
			*Chrysoperla* sp. 2	●	●	●
			*Chrysoperla* sp. 3		●	
	** Mantispidae **	Mantispinae	Mantispinae gen sp. 1	●	●	●
			Mantispinae gen sp. 2		●	
** Odonata **						
	** Chlorocyphidae **	Calopteryginae	*Libellago* sp.		●	
	** Coenagrionidae **	Pseudostigmatinae	*Enallagma* sp.	●	●	
** Orthoptera **						
	** Acrididae **		*Alolopus thalassinus*		●	
	** Gryllidae **	Gryllinae	Gryllinae gen. sp. 1	●		
			Gryllinae gen. sp. 2	●		
			Gryllinae gen. sp. 3	●	●	●
		Oecanthinae	Oecanthinae gen. sp.		●	
		Trigonidiinae	*Anixipha* sp.	●	●	●
** Thysanoptera **						
	** Phlaeothripidae **		Phlaeothripidae gen. sp. 1	●		●
			Phlaeothripidae gen. sp. 2	●	●	

**Table 2.** List of flora species in riparian zones of Sungai Sepetang, Sungai Rembau and Sungai Chukai, Peninsular Malaysia

**Family**	**Species**	**Sungai Sepetang**	**Sungai Rembau**	**Sungai Chukai**
Anacardiaceae	*Parishia insignis*	0	1	0
Apocynaceae	*Cerbera odollam*	0	2	9
Arecaceae	*Nypa fruticans*	15	13	4
Arecaceae	*Oncosperma tigillarium*	0	0	2
Asteraceae	*Mikania micrantha*	0	2	0
Fabacea	*Caesalpinia crista*	1	5	3
Lamiaceae	*Vitex pinnata*	0	0	1
Lecythidaceae	*Barringtonia racemosa*	0	1	18
Lythraceae	*Sonneratia caseolaris*	19	19	13
Malvaceae	*Hibiscus tiliaceus*	0	6	8
Melastomataceae	*Melastoma malabathricum*	0	0	1
Moraceae	*Ficus microcarpa*	1	0	0
Pteridaceae	*Acrostichum aureum*	8	3	1
Pteridaceae	*Acrostichum speciosum*	4	0	0
Rhizophoraceae	*Rizophora apiculata*	6	1	1
Rhizophoraceae	*Bruguiera sexangula*	0	0	1

**Table 3.** Summary of ANOVA results for Sungai Sepetang, Sungai Rembau and Sungai Chukai. Mean values of each environmental parameter are given.

	**F**	**df**	**p**	**Sg. Sepetang**	**Sg. Rembau**	**Sg. Chukai**
Wind speed (m/s)	17.39	2	P < 0.05	0.39 ± 0.30	0.56 ± 0.24	0.38 ± 0.16
Salinity (NaCl %)	35.77	2	P < 0.05	2.94 ± 1.50	1.04 ± 1.02	0.21 ± 0.04
Temperature (⁰C)	144.55	2	P < 0.05	27.68 ± 1.88	29.89 ± 0.73	26.17 ± 0.41
Relative Humidity (%)	69.87	2	P < 0.05	75.74 ± 13.14	70.58 ± 3.81	79.82 ± 1.19
Light intensity (lux)	34.75	2	P < 0.05	64.33 ± 6.60	34.59 ± 15.72	35.58 ± 7.66

**Table 4.** Spearman’s correlation value between abundance, richness and diversity of insect to selected abiotic factors. Numbers in bold indicate correlation significant at 0.05 level.

	**Sungai Sepetang**		**Sungai Rembau**		**Sungai Chukai**	
**Abiotic factors**	**Abundance** **(Number of individuals)**	**Diversity** **(H’)**	**Abundance** **(Number of individuals)**	**Diversity** **(H’)**	**Abundance** **(Number of individuals)**	**Diversity** **(H’)**
Temperature	0.112	-0.671	0.112	-0.447	-0.359	-0.616
Humidity	-0.447	0.447	0.112	-0.783	0.667	-0.103
Wind Speed	-**0.447**	-**0.266**	-0.200	-0.100	0.359	-0.410
Salinity	-0.089	-**0.454**	0.298	0.146	-**0.465**	-0.422
